# Brachial Blood Pressure Invasively and Non-Invasively Obtained Using Oscillometry and Applanation Tonometry: Impact of Mean Blood Pressure Equations and Calibration Schemes on Agreement Levels

**DOI:** 10.3390/jcdd10020045

**Published:** 2023-01-26

**Authors:** Daniel Bia, Yanina Zócalo, Ramiro Sánchez, Juan F. Torrado, Gustavo Lev, Oscar Mendiz, Franco Pessana, Agustín Ramírez, Edmundo I. Cabrera-Fischer

**Affiliations:** 1 Departamento de Fisiología, Facultad de Medicina, Centro Universitario de Investigación, Innovación y Diagnóstico Arterial (CUiiDARTE), Universidad de la República, Montevideo 11800, Uruguay; 2 Metabolic Unit and Hypertension Unit, University Hospital, Favaloro Foundation, Buenos Aires 1093, Argentina; 3 Department of Interventional Cardiology, University Hospital, Favaloro Foundation, Buenos Aires 1093, Argentina; 4 Department of Information Technology, Engineering and Exact Sciences Faculty, Favaloro University, Buenos Aires 1746, Argentina; 5 IMETTYB, Favaloro University—CONICET, Buenos Aires 1746, Argentina

**Keywords:** aortic pressure, applanation tonometry, brachial blood pressure, calibration procedure, invasive records, mean blood pressure, non-invasive records, oscillometry, pulse waveform

## Abstract

The use of oscillometric methods to determine brachial blood pressure (bBP) can lead to a systematic underestimation of the invasively measured systolic (bSBP) and pulse (bPP) pressure levels, together with a significant overestimation of diastolic pressure (bDBP). Similarly, the agreement between brachial mean blood pressure (bMBP), invasively and non-invasively measured, can be affected by inaccurate estimations/assumptions. Despite several methodologies that can be applied to estimate bMBP non-invasively, there is no consensus on which approach leads to the most accurate estimation. Aims: to evaluate the association and agreement between: (1) non-invasive (oscillometry) and invasive bBP; (2) invasive bMBP, and bMBP (i) measured by oscillometry and (ii) calculated using six different equations; and (3) bSBP and bPP invasively and non-invasively obtained by applanation tonometry and employing different calibration methods. To this end, invasive aortic blood pressure and bBP (catheterization), and non-invasive bBP (oscillometry [Mobil-O-Graph] and brachial artery applanation tonometry [SphygmoCor]) were simultaneously obtained (34 subjects, 193 records). bMBP was calculated using different approaches. Results: (i) the agreement between invasive bBP and their respective non-invasive measurements (oscillometry) showed dependence on bBP levels (proportional error); (ii) among the different approaches used to obtain bMBP, the equation that includes a form factor equal to 33% (bMBP = bDBP + bPP/3) showed the best association with the invasive bMBP; (iii) the best approach to estimate invasive bSBP and bPP from tonometry recordings is based on the calibration scheme that employs oscillometric bMBP. On the contrary, the worst association between invasive and applanation tonometry-derived bBP levels was observed when the brachial pulse waveform was calibrated to bMBP quantified as bMBP = bDBP + bPP/3. Our study strongly emphasizes the need for methodological transparency and consensus for non-invasive bMBP assessment.

## 1. Introduction

Previous reports have postulated that brachial artery blood pressure (bBP) determination using non-invasive methods could (in general terms) underestimate systolic (bSBP) and pulse (bPP) brachial pressure invasively obtained, at the time diastolic brachial blood pressure (bDBP) would be overestimated [1,2,3]. These inaccuracies could impact on the association between bBP levels and cardiovascular health and disease, and thus, on the capability to understand cardiovascular physiology and pathophysiology. On the other hand, it could lead to inaccurate brachial artery mean blood pressure (bMBP) estimations. Additionally, when using bBP data to calibrate non-invasive aortic blood pressure (aoBP) recordings, the above could also affect the determination of aoBP absolute levels, and/or the comparative analysis between aoBP and bBP, which would be ofimportant experimental and clinical relevance [1,4,5,6,7,8,9,10,11]. Consequently, although widely used, the non-invasive determination of bBP itself requires further research.

In this context, at least two issues deserve further investigation: On the one hand, analyzing to what extent the agreement (equivalence) between invasive and non-invasive bSBP, bPP and bMBP recordings (obtained with oscillometry) could depend on the bBP levels considered (proportional error) and/or on the method used to obtain bMBP (direct by oscillometry vs. indirect, using different equations). On the other hand, it remains to be identified to what extent the levels of agreement (equivalence) between bBP levels obtained invasively and those obtained non-invasively by applanation tonometry could depend on the calibration scheme considered. In this regard, multiple calibration approaches have been proposed and used (e.g., based on bSBP/bDBP or bMBP/bDBP) [1,4,5,6,7,8,9,10,11].

Up to now, it has been accepted that a more accurate estimate of invasive blood pressure is obtained when calibrating to bMBP/bDBP values rather than to bSBP/bDBP [10,11]. On the other hand, it is recognized that when using the bMBP/bDBP calibration scheme, a major limitation (source of bias) is bMBP estimation itself, as the only way to obtain an accurate and reliable measurement of “real” bMBP is by means of intra-arterial recordings (catheterization). Regardingthis, several arithmetic and mathematic integration methods have been proposed to calculate bMBP [11] and there is no current consensus on which yields the most accurate bMBP estimate. An additional unsolved matter is to what extent “real” mean blood pressure (MBP) can be considered unchanged throughout great arteries, and thus, whether bMBP and aortic mean blood pressure (aoMBP) are similar. All these issues require further validation research aimed at determining the accuracy of different formulas to calculate bMBP, by comparing them with invasive bMBP and aoMBP [11].

In this context, the aims of this study were: First (Aim 1), to invasively assess bBP and aoBP levels and waveforms and to evaluate the agreement between them, in terms of systolic, diastolic, pulse and, mainly, MBP levels (aoMBP vs. bMBP). Second (Aim 2), to evaluate the association and agreement between bSBP, bPP and bDBP values invasively and non-invasively obtained, using oscillometric measurements. Third (Aim 3), to analyze the association and agreement between bMBP values invasively obtained and those (i) measured (oscillometry) and (ii) calculated using six methods (formulas) that employ bSBP and bDBP non-invasively measured, using an oscillometric device. Finally (Aim 4), to evaluate the association and agreement between bSBP and bPP values invasively assessed and those non-invasively obtained using applanation tonometry in the brachial artery, employing different calibration methods.

## 2. Materials and Methods

### 2.1. Subjects

Thirty-four subjects (41% females; 14–89 years-old) with coordinated coronary angiogram at the Favaloro Foundation University Hospital were included [12]. Subjects with valvular heart disease and/or arrhythmia were excluded. A brief clinical interview, together with an anthropometric evaluation, enabled us to assess the exposure to cardiovascular risk factors (CRFs), defined according to criteria previously described [13,14,15,16,17]. Body weight (Omron HBF-514C, Omron Healthcare, Inc., Lake Forest, IL, USA) and height (portable stadiometer) were measured with the participants wearing light clothing and no shoes. Laboratory biochemical data were obtained, and echocardiographic examinations were performed (Table 1).

**Figure 1:**
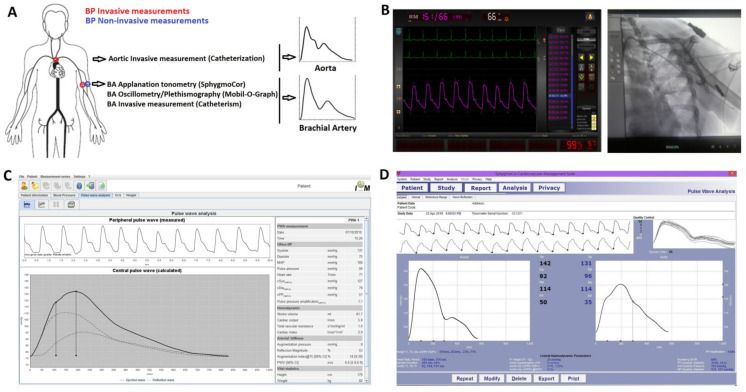
Schematic representation of invasive and non-invasive aortic and brachial blood pressures (BP) measurements (**A**). Invasive brachial recordings (**B**). Non-invasive approaches using oscillometry (**C**) and applanation tonometry (**D**).

A specialized nurse gave patients general explanatory guidance on the invasive procedure to be performed. Prior to the evaluation, written informed consent was obtained from the participants and/or their parents. Informed assent was obtained when necessary. The signed document specified that the following measurements would be performed: (a) vascular catheterization to measure aoBP and bBP levels and waveforms, and (b) non-invasive measurement of bBP levels and waves using applanation tonometry and oscillometry/plethysmography (Figure 1A). The study was performed without changes in the pharmacological therapy.

### 2.2. Invasive Aortic and Brachial Blood Pressure Recordings

#### 2.2.1. Central Aortic Blood Pressure

Intra-arterial aoBP and bBP levels and waveforms were obtained with the subjects lying in the supine position, according to routine clinical practice and guidelines for invasive coronary arteries assessment. Briefly, standard asepsis was performed in the arterial access area (radial), followed by a cutaneous/subcutaneous injection of lidocaine to minimize the patient’s pain and discomfort. A soft sedation (midazolam at 1.5 mg and fentanyl at 0.025 mg) was also administered as needed. After local anesthesia was applied in the vessel access area, a 5- or 6-French introducer sheath was positioned in the arterial lumen, and heparin (5000 units) was administered through the arterial catheter. Subsequently, a 0.035-inch guide wire was advanced and placed in the ascending aorta, and a 5-French pigtail catheter (Cordis, Miami, FL, USA) was introduced thereafter. Special attention was paid to place the tip of the pigtail catheter ~4 cm away from the aortic valve. After confirming the correct positioning of the catheter, which was assessed visually via fluoroscopy (Allura Xper FD10 or AlluraClarity FD20/10, Philips Healthcare, Amsterdam, The Netherlands), the guide wire was removed, and the intra-arterial catheter was flushed with saline solution.

To record intravascular pressure, the aforementioned fluid-filled catheter placed in the proximal ascending aorta (or brachial artery) was connected to the external blood pressure transducer (MX960, Medex, LogiCal, Smiths Medical ASD Inc., Minneapolis, MN, USA), and the transducer was connected to the AcistCVi system (AcistCVi, Medical System Inc., Heerlen, The Netherlands). The MX960 meets (or exceeds) the specifications (statements) of the Association for the Advancement of Medical Instrumentation/European Society of Hypertension/International Organization for Standardization (AAMI/ESH/ISO) Collaboration. The AsistCVi system was synchronized with the X-ray imaging system Allura Xper FD10 or AlluraClarity FD20/10 (Philips Healthcare, The Netherlands).

Prior to each measurement, the combined system of catheter, tubing and external transducer was flushed with saline solution, and the aoBP (or bBP) trace was visually inspected for quality. According to the calibration scheme recommended by the manufacturer, the external pressure transducer was calibrated following the system’s inbuilt 2-point calibration method. First, “zero”was assigned to the pressure value recorded when the sensor was opened to the atmosphere (adjusting the baseline to zero or atmospheric pressure), and second, by exposing the transducer to a pressure level equal to 100 mmHg (the device itself exposes the transducer to 100 mmHg, and the operator checks that it is the pressure level displayed on the recording monitor). In the Cardiac Catheterization Laboratory of the Favaloro Foundation University Hospital, the dynamic response of the catheter, tubing and external transducer combined system was adjusted to ensure a: (i) a natural frequency of at least 20 Hz and (ii) a damping coefficient of at least 0.3. It was demonstrated that several external transducers, including the one used in the present work (MX960, LogiCal), have a high quality, distortion-free frequency response within the bandwidth of 0 to 30 Hz [18]. The quantitative limits described above guarantee an adequate compromise between the natural frequency and the damping coefficient, which ensures that measurement systems operate in areas of adequate dynamic responses or, at worst, in a very slightly under-damped region. The external transducer was maintained at heart (mid-axillary line) level. Invasive blood pressure waveforms were visualized in the Allura Xper FD10 or AlluraClarity FD20/10 monitor images (Philips Healthcare, The Netherlands).

Intra-arterial aoBP levels and waves were recorded (for at least 45 s), together with the respective non-invasive bBP measurements (oscillometry and applanation tonometry) obtained in the contra-lateral arm (see below).

#### 2.2.2. Brachial Artery Blood Pressure

Once the invasive aoBP recordings were obtained, the catheter was positioned in the contra-lateral brachial artery at the level where the pneumatic cuff for non-invasive pressure measurement was located (Figure 1B). Thereafter, intra-arterial bBP levels and waveforms were recorded (for at least 45 s), together with their respective non-invasive bBP measurements (oscillometry and applanation tonometry).

Thus, at recording, triads were obtained (at least three with invasive aoBP and three with invasive bBP recordings), and each one was composed by: (i) an invasive recording (for at least 45 s), and a non-invasive recording using (ii) brachial artery oscillometric/plethysmography (Mobil-O-Graph device) and (iii) brachial artery applanation tonometry (SphygmoCor device).

Following each invasive and non-invasive bBP recording, the 5-French catheter was re-positioned in the aorta and additional aoBP levels and waveforms were obtained. The comparison of aortic recordings obtained before and after the brachial recordings allowed us to assess the hemodynamic stability of the subject. The beat-to-beat levels of systolic, diastolic, MBP (i.e., area under the pressure/time curve, divided by the cardiac cycle time) and heart rate (HR) were determined by means of data analysis processing systems.

### 2.3. Non-Invasive Brachial Artery Blood Pressure Measurement

As mentioned, two different non-invasive devices (techniques) were used to assess bBP waveforms [7,16] (Figure 1C,D). Oscillometric/plethysmographic bBP levels and waveforms recordings were obtained with the Mobil-O-Graph automatic device (Model PWA, IEM GmbH, Stolberg, Germany). To this end, a pneumatic cuff properly sized according to the patient characteristics [7,16] was positioned in the arm (in our case, the contralateral to be used for the sheath insertion) (Figure 1C). Then, HR and bMBPosc (point of lowest bBP corresponding to the maximal oscillations) were registered, and bSBPosc and bDBPosc were obtained by means of the internal algorithms of the device manufacturer. At least six non-invasive recordings were obtained simultaneously, and immediately before and/or after invasive aoBP and bBP measurements. Only high-quality records (index equal to 1 or 2) and satisfactory waveforms (visual inspection) were considered for the data analysis [7,16]. For each subject, the bBP value reported is the average of the records obtained in each determination.

The brachial BP waveforms and values were assessed using applanation tonometry (SphygmoCorCvMS; AtCorMedical, Sydney, Australia), before and/or after oscillometric/plethysmographic bBP measurements, and simultaneously with invasive aoBP or bBP recordings (Figure 1D). Applanation tonometry provides beat-to-beat BP waves (10 s) [7,16] that can be calibrated with different calibration schemes (see below).

Once the invasive aoBP and bBP and non-invasive bBP measurements concluded, the catheter was removed, and each subject was taken to the recovery area. Once the subject’s clinical condition was considered stable, the patient was discharged from the hospital. No collateral harm or complications were observed during the evaluations.

### 2.4. Mean Blood Pressure Quantification

Oscillometry-derived (Mobil-O-Graph) bSBP, bDBP, bPP and HR data were named ’bSBPosc’, ‘bDBPosc’, ‘bPPosc’ and ‘HRosc’, respectively (Table 2). In turn, the bMBP level obtained with oscillometry was identified as ‘bMBPosc’. Then, using the bSBPosc and bDBPosc values, bMBP was quantified as described below [11] (Table 2):

(i) bMBP_0.42_ [mmHg] = 0.42*bSBP_osc_ + 0.58 × bDBP_osc_
(ii) bMBP_0.412_ [mmHg] = bDBP_osc_ + [0.412 × (bSBP_osc_ − bDBP_osc_)]
(iii) bMBP_0.33_ [mmHg] =bDBP_osc_ + 0.33 × (bSBP_osc_ − bDBP_osc_)
(iv) bMBP_+5_ [mmHg] = bDBP_osc_ + [0.33 × (bSBP_osc_-bDBP_osc_) + 5]
(v) bMBP_0.33HR_ [mmHg] =bDBP_osc_ + [0.33 + (0.0012 × HR_osc_)] × (bSBP_osc_ − bDBP_osc_)
(vi) bMBP_SBP×DBP_^0.5^ [mmHg] = (bSBP_osc_ × bDBP_osc_)^0.5^

### 2.5. Calibration of the Tonometry-Derived Signals

The bBP waveforms obtained using applanation tonometry (AT) were calibrated as follows (Table 2):

1. Invasive-derived (‘Inv’): the invasively obtained bMBP and bDBP (arterial catheterization) were, respectively, assigned to the algebraic mean and minimum of the brachial artery wave. Then, the obtained bSBP, bDBP and bMBP were called: ‘bSBP(AT_Inv)’, ‘bDBP (AT_Inv)’ and ‘bMBP(AT_Inv)’, respectively.
2. Systo-diastolic (‘SD’): bSBPosc and bDBPosc were, respectively, assigned to the maximum and minimum of the brachial artery wave. Then the bSBP, bDBP and bMBP values were called: ‘bSBP(AT_SD)’, ‘bDBP(AT_SD)’ and ‘bMBP(AT_SD)’, respectively.
3. Oscillometric-derived (‘Osc’): bMBP_osc_ and bDBP_osc_ were, respectively, assigned to the algebraic mean and minimum brachial wave. Then, the obtained bSBP, bDBP and bMBP were called: ‘bSBP(AT_Osc)’, ‘bDBP(AT_Osc)’ and ‘bMBP(AT_Osc)’, respectively.
4. Calculated MBP: bDBP_osc_ and bMBP (calculated from oscillometry-derived bSBP_osc_ and bDBP_osc_, using different equations [bMBP_0.412_; bMBP_0.33_; bMBP_0.33HR_]) were, respectively, assigned to the minimum and to the algebraic mean of the brachial wave. Then, bSBP, bDBP and bMBP were called:
  (i) ‘bSBP(AT_033)’, ‘bDBP(AT_033)’, ‘bMBP(AT_033)’;
  (ii) ‘bSBP(AT_033HR)’, ‘bDBP(AT_033HR)’, ‘bMBP(AT_033HR)’;
  (iii) ‘bSBP(AT_0412)’, ‘bDBP(AT_0412)’, ‘bMBP(AT_0412)’.

### 2.6. Data and Statistical Analysis

A stepwise data analysis was carried out taking into account the aims of this work. To this end, Lin’s Concordance Correlation Coefficient (CCC), Intraclass Correlation Coefficient (ICC) and/or Bland–Altman (considering unique [n = 34] and multiple or repeated [n = 193] measurements) analyses were conducted.

First, the association and agreement between invasive bBP and aoBP levels (in terms of SBP, DBP, PP and MBP) were quantified. (Table 3).

Second, the association and agreement between invasive and oscillometry-derived non-invasive bBP data in terms of SBP, DBP and PP were quantified (Table 4).

**Figure 2:**
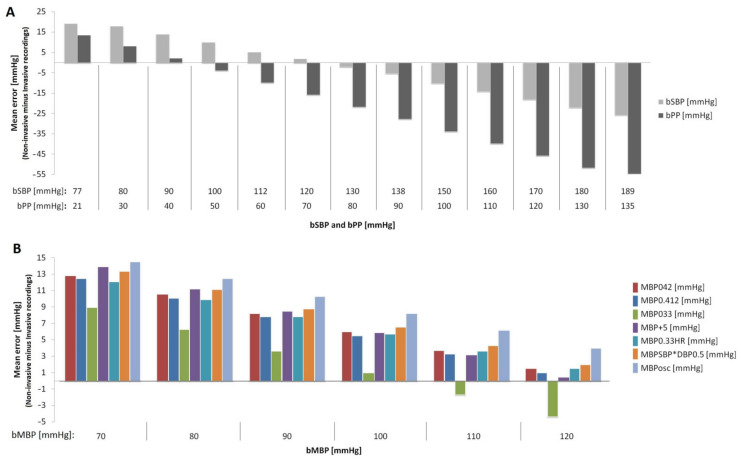
(**A**): Errors between invasive and oscillometric bSBP and bPP data for different BP levels. (**B**): Errors between invasive bMBP data and non-invasive bMBP data obtained considering several calibration schemes and different BP levels. HR: heart rate. Osc: oscillometric measurement.

Third, by applying a similar statistical approach, the association and agreement between invasive bMBP and non-invasive bMBP were assessed, considering the different methods used to measure or quantify bMBP: (i) bMBP_osc_, (ii) bMBP_0.42_, (iii) bMBP_0.412_, (iv) bMBP_0.33_, (v) bMBP_+5_, (vi) bMBP_0.33HR_ and (vii) bMBP_SBP*DBP_^0.5^(Table 4). Considering the mean and proportional (regression equation) errors that arose from the Bland–Altman analysis, the difference between the invasive and non-invasive (oscillometry) data was calculated for different bSBP, bPP and bMBP values (Figure 2).

**Figure 3:**
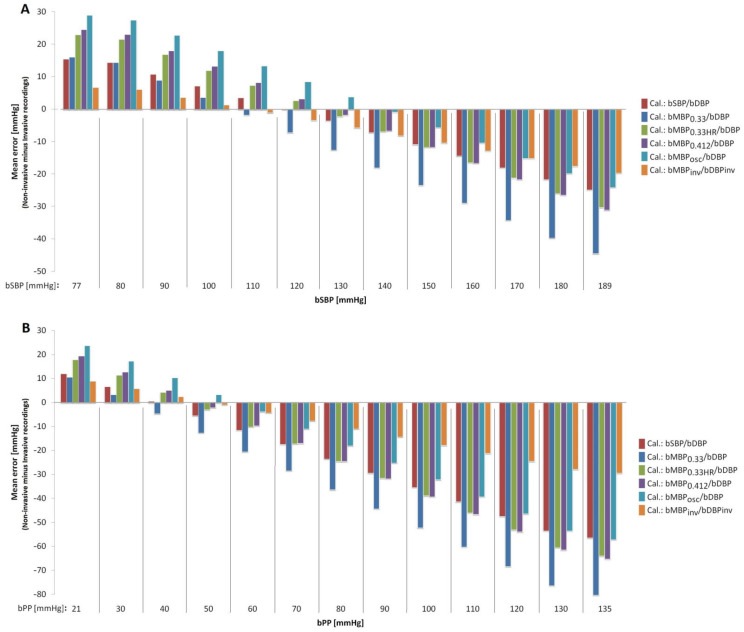
(**A**): Errors between non-invasive brachial systolic blood pressure (bSBP) obtained using different calibration schemes and invasive bSBP data (for different pressure levels). (**B)**: Errors between non-invasive pulse pressure (bPP) obtained with different calibration schemes and invasive bPP measurements (for different BP levels).

Fourth, the association and agreement between invasive and tonometry-derived bSBP and bPP were analyzed considering the different calibration schemes (Table 5). From the mean and proportional (regression equation) errors producedby the Bland–Altman analysis, the difference between the invasive and non-invasive (tonometry-derived) data was calculated for the different bSBP (Figure 3A) and bPP (Figure 3B) values.

In all cases, the Bland–Altman plots correspond to the mean of the data obtained with the different methods (e.g., invasive and non-invasive bSBP, x-axis) versus their difference (e.g., invasive bSBP minus non-invasive bSBP, y-axis). The corresponding linear regression equations were obtained. Systematic error (bias) was considered present if the mean error was significantly different from zero, whereas proportional error was considered present if the slope of the linear regression was statistically significant. Statistical analyses were conducted considering per subject: (i) a pair of measurements (n = 34, Bland–Altman classic or original test) and (ii) more than a pair of measurements (n = 193, Bland–Altman test for repeated measurements).

According to the central limit theorem, taking into account the kurtosis and skewness coefficients distribution and the number of subjects (sample size >30), a normal distribution was considered [19]. Data analyses were performed using MedCalc (v.14.8.1, MedCalc Inc., Ostend, Belgium) and IBM-SPSS Statistical Software (v.26, SPSS Inc., Chicago, IL, USA). A *p* value < 0.05 was considered statistically significant.

## 3. Results

### 3.1. Population and Hemodynamic Characteristics

The general characteristics of the studied subjects are shown in Table 1. The group was characterized by a wide age range (61 ± 19 y, range: 14–89 y) with good sex distribution (Table 1).

Descriptive information on the hemodynamic characteristics can be seen in Table 2. The invasive bSBP values were distributed in a wide range: 6.5% of the analyzed population had < 100 mmHg, 58.1% between 100 and 139 mmHg, 19.4% between 140 and 159 mmHg, and 16.1% exhibited values ≥ 160 mmHg. On the other hand, the distribution of invasive bDBP values was 19.3% < 60 mmHg, 71.0% between 60 and 84 mmHg, and 9.7% > 85 mmHg. The HR values were always within a normal range.

### 3.2. Agreement between Invasive Aortic and Brachial Blood Pressure (Aim 1)

The invasive SBP, MBP, DBP and PP measured in the brachial artery and aortic root showed a straight (simple bivariate) correlation (r range: 0.86–0.90) (Table 3). However, aortic and brachial recordings were not equivalent in any comparison including SBP, MBP, DBP and PP (CCC range: 0.78–0.87; ICC range: 0.79–0.88) (Table 3). The invasive bBP values were almost always higher than those measured in the aortic root (SBP: 146 ± 5 vs. 135 ± 4 mmHg, *p* < 0.01; DBP: 71 ± 2 vs. 68 ± 2 mmHg, *p* < 0.01; PP: 75 ± 5 vs. 67 ± 4 mmHg, *p* < 0.01; MBP: 98 ± 3 vs. 94 ± 3 mmHg, *p* < 0.01) (Table 2).

For invasive SBP and PP readings, the mean error of the difference (brachial minus aorta) was on average 10.46 and 8.17 mmHg, respectively (Table 3). It is worth noting that there were subjects whose aortic pressure levels were higher than the brachial values. Regardingthis, the 95% CI for the differences (brachial minus aorta) was 37.29 to −16.36 mmHg for SBP and 33.60 to −17.25 mmHg for PP (values represent the 95% CI upper and lower limit, respectively) (Table 3). No proportional error was observed (non-significant *p* of the slope) (Table 3). In other words, the differences between bBP and aoBP were not associated with the BP level itself (aortic and brachial BP mean).

On average, the mean errors for MBP and DBP were 3.79 mmHg (95%CI: 1.5 to 6.1 mmHg) and 2.31 mmHg (95% CI: 0.4 to 4.2 mmHg), respectively. Again, the observed differences were not associated with the BP level itself, meaning that there was no proportional error (non-significant p of the slope) (Table 3). The 95% CI (upper and lower limit, respectively) for the differences (brachial minus aorta) ranged between 16.22 and −8.63 mmHg for invasive MBP and between 12.54 and −7.93 mmHg for DBP data. As previously described, classic and repeated measurements Bland–Altman analyses were performed. As can be seen in Table 3, their results were similar.

### 3.3. Agreement between Invasive and Non-Invasive (Oscillometric) Brachial Blood Pressure (Aim 2)

The invasive bSBP, bPP and bDBP values were significantly associated with the corresponding non-invasive oscillometry-derived (Mobil-O-Graph) data (simple bivariate correlation, r: 0.88, 0.70 and 0.58, respectively, *p* < 0.001) (Table 4). However, the invasive and oscillometric data were not equivalent in any comparison (CCC: 0.77, 0.39 and 0.42 for bSBP, bDBP and bPP, respectively) (Table 4). The invasive bSBP and bPP values were always higher than those obtained with oscillometry (SBP: 146 ± 5 vs. 137 ± 3 mmHg, *p* < 0.01; PP: 75 ± 5 vs. 56 ± 3 mmHg, *p* < 0.01). Conversely, the invasive bDBP values were lower than those obtained with oscillometry (DBP: 71 ± 2 vs. 81 ± 2 mmHg, *p* < 0.01) (Table 2).

The non-invasive bSBP and bPP values (arithmetic mean or systematic error) were 8.2 mmHg and 18.8 mmHg lower, respectively, than those obtained invasively (Table 4). However, these differences were BP-dependent, since invasive and non-invasive bSBP and bPP data showed proportional errors (Table 4, Figure 2A). The invasive bSBP levels were over-estimated by oscillometric measurements within the range 77–125 mmHg (Figure 2A). On the contrary, the invasive bSBP values were underestimated by the oscillometry within 125–189 mmHg (Figure 2A). It is worth noting that the mean (systematic) error of the oscillometric recordings was <5 mmHg when the invasive bSBP levels were in between 112 and 138 mmHg (Figure 2A). On the other hand, the invasive bPP levels within a range of 21–44 mmHg were overestimated by the non-invasive oscillometric recordings, while the invasive bPP values between 44 and 135 mmHg were underestimated by the non-invasive data (Figure 2A). Although the non-invasive bDBP values obtained with the oscillometric device were on average 10.6 mmHg higher than those obtained invasively, there were no proportional biases (Slope, *p* = 0.158; differences were not associated with BP levels) (Table 4).

### 3.4. Mean Blood Pressure: Agreement between Invasive and Non-Invasively Derived (Measured and Calculated) Values (Aim 3)

The bMBP levels quantified by different equations or directly measured with oscillometry showed positive associations with invasive bMBP (simple bivariate correlation, r range: 0.80 to 0.83), but they were not equivalent (CCC: 0.70–0.80; ICC: 0.71–0.80). Moreover, both systematic and proportional errors were identified (Table 4). The mean errors between non-invasive and invasive bMBP were between 1.4 mmHg for bMBP_0.33_ (non- significant) and 8.5 mmHg for bMBP_osc_ (Table 4). The biases for the bMBP data obtained from non-invasive oscillometric recordings (and considering different calibration schemes) were, in general terms, higher at lower bMBP levels (proportional errors, Figure 2B).

The equation bMBP = bDBP(Osc) + bPP(Osc) × 0.33 (form factor = 33%) was the only one that derived bMBP without statistically significant differences with respect to the invasive bMBP data (mean error: 1.4 mmHg, 95% CI: −1.8 to 4.7 mmHg, *p* = 0.374) (Table 4). On the other hand, when the form factor 33% (bMBP_033_) was considered, the mean error was 8.84 mmHg for invasive bMBP levels equal to 70 mmHg and −4.26 mmHg for invasive bMBP levels equal to 120 mmHg, which indicates the existence of proportional (pressure-dependent) bias (Table 4; Figure 2B). It is important to consider that the use of a form factor of 33% in the bMBP formula would minimize the differences with respect to invasive bMBP, except for values in the upper limit (≥120 mmHg) (Figure 2B).

Disregarding the bMBP levels considered, the data from oscillometry (bMBPosc) showed the greatest differences with respect to invasive bMBP [Figure 2B]. Here, the mean error was 8.5 mmHg. In addition, for bMBP values equal to 70 and 120 mmHg, the biases between the invasive and bMBPosc data were 14 and 4 mmHg, respectively (Table 4; Figure 2B).

The bMBP values obtained with other equations showed similar statistically significant mean errors (~6 mmHg), but the equation that uses a form factor of 33% and is corrected for HR (bMBP_033HR_) was the only one without proportional bias (*p* = 0.0508, umbral of significance) (Table 4; Figure 2B).

Similar findings were observed when analyses were carried out using a pair of measurements per subject and when more measurements were considered (Table 4).

### 3.5. Brachial Blood Pressure Values Obtained with Applanation Tonometry Using Invasive and Non-Invasive Calibration (Aim 4)

Table 2 (lower half) shows the invasive hemodynamic data obtained for the brachial artery and the ascending aorta (top), together with the non-invasive BP values obtained with oscillometry (middle) applied to re-calibrate brachial artery tonometry-derived signals (bottom). Note that the bSBP values derived from tonometry using different calibration schemes showed a wide variation (i.e., bSBP between 124 and 142 mmHg) (Table 2).

Table 5 and Figure 3 show the comparative analysis of invasive and tonometry-derived bBP levels. Invasively and non-invasively obtained brachialSBP and PP data (tonometry) showed significant positive simple bivariate associations (except for bPP_033_). However, the association level (strength) varied depending on the calibration method (r range: 0.67 to 0.89 for bSBP, and 0.39 to 0.70 for bPP). CCC and ICC analyses showed that the invasive and tonometry-derived data were far from being equivalent (Table 5).

Bland–Altman’s analyses showed that, although all the calibration methods underestimated (mean error) invasive bSBP, the calibration based on thebMBP_osc_ values allowed us to minimize the differences between the invasive and non-invasive data (bSBP mean bias = −4.0 mmHg, bPP mean bias = −15.4 mmHg) (Table 5). On the contrary, the use of the bMBP_033_-based calibration method showed the greatest differences (bSBP mean error = −21.6 mmHg, and bPP mean error = −33.0 mmHg).

In summary, all the calibration methods showed proportional errors (Table 5, Figure 3). Figure 3 shows that, regardless of the calibration scheme considered, the non-invasive BP methods changed from overestimating to underestimating the BP values as the bSBP and bPP levels increased. The differences between invasive and non-invasive bSBP were minimized when invasive bBP was used as the calibration method, with differences <5 mmHg for bSBP within the range 85–125 mmHg (Figure 3A). However, the differences increased when higher bSBP values were considered (e.g.: for bSBP = 190 mmHg the mean error was ~−20 mmHg) (Figure 3A). Similar findings were observed for bPP (Table 5; Figure 3B).

## 4. Discussion

### 4.1. Agreement between Invasive Brachial and Aortic Blood Pressure (Aim 1)

First, invasive brachial and aortic DBP and MBP showed differences. On average (mean error), the MBP and DBP values were, respectively, ~4 mmHg (95% CI: 1.5–6.1 mmHg) and ~2 mmHg (95% CI: 0.4–4.2 mmHg) higher in the brachial artery (Table 3). Althoughit is generally accepted that aortic and brachial MBP and DBP were similar when a subject was supine in our study, there were differences between central and peripheral data. Regarding this, there were subjects whose aoMBP was higher than the brachial, and others whose bMBP was higher. This is an important finding, as the constancy of the DBP and MAP values along the great arteries (when a subject is supine) is widely accepted and frequently used in the development of hemodynamic models applied to characterize and understand physiological and pathophysiological phenomena of the arterial system. In fact, the assessment of aoBP by most of the non-invasive methods is based on the described hemodynamic assumption (identical MBP and DBP in the aorta and brachial artery). Although the mean difference in MBP was only 3–4 mmHg (with a 95% CI for the mean error between 1.47 and 6.12 mmHg), 95% of the readings werewithin the range of differences between −8.63 and 16.22 mmHg (Table 3). Consequently, there were subjects in whom it would be clearly inaccurate to assume that the MBP (and DBP) values obtained peripherally or centrally weresimilar. Previous studies showed data (trends) similar to the described in this work. In subjects (n = 40) referred for diagnostic coronary catheterization, Shih et al. reported that bMBP was significantly higher than aoMBP (simultaneous measurements with custom-made dual pressure sensor catheter) [20]. Additionally, Nakaomi et al. (2017) reported invasive BP measurements (fluid-filled catheter) in patients who underwent elective coronary angiography. The authors found that bDBP values were on average 1 mmHg higher than those registered in the aorta (they did not perform statistical comparisons) [21]. Further studies must be performed to clarify the issue and to identify the explanatory factors for the differences between the central and peripheral BP data. In this regard, future work should analyze whether the (unexpected) findings mentioned above are associated with specific biological characteristics of the cardiovascular system of the studied subjects and/or with technical factors related to the methodological approach considered (e.g., the height of the hydrostatic column in the external pressure transducer would be different when the catheter tip is placed in the aorta vs. in the brachial).

On the other hand, SBP levels tended to be higher in the brachial artery than in the aorta (Table 2 and Table 3), which is in agreement with the known SBP and PP peripheral amplification phenomenon. In turn, the differences were reduced in older subjects, which is consistent with BPb being considered “more representative” (more alike) aoBP.

Finally, it is worth mentioning that the differences observed between bBP and aoBP were not influenced by BP levels (no proportional error was detected) (Table 3). This observation would “simplify the situation”, based onthe understanding that potential models that could correct the calibration methods considering the differences in the levels of DBP and MBP between the brachial artery and aorta should not consider the specific BP data.

### 4.2. Agreement between Invasive and Non-Invasive (Oscillometric) Brachial Blood Pressure (Aim 2)

Second, the oscillometric-derived bSBP and bPP values (mean error) were, respectively,~8 mmHg and ~19 mmHg lower than those obtained invasively. In addition, proportional errors were observed (Table 4, Figure 2). Invasive bSBP levels within the rangeof 77–125 and 125–189 mmHg were overestimated and underestimated by non-invasive oscillometric measurements, respectively (Figure 2A). Only volunteers in whom bSBP was in the range of 112–138 mmHg had a calculated error <5 mmHg (Figure 2A). On the other hand, invasive bPP levels within 21–44 and 44–135 mmHg were, respectively, overestimated and underestimated by non-invasive oscillometric measurements, respectively. In turn, the non-invasive oscillometric bDBP values were, on average, ~11 mmHg higher than those obtained invasively, but no proportional error was observed (Table 4). Thus, this difference of about 8 (for SBP) and 19 mmHg (for PP) suggests that this misestimation of bSBP and bPP could significantly impact the accurate estimate of the cardiovascular risk in the clinical practice.

This result adds to previous reports [1,2,3] in which the investigators found, on average, that non-invasive SBP and DBP data underestimated and overestimated the invasive BP, respectively. The most important finding, however, was the different situations in terms of BP “over- and underestimation” depending on bBP levels. Undoubtedly, “the worst” situation would be when the real (invasive) bBP is underestimated, which would lead to under diagnosis, a lack of treatment initiation and inaccurate cardiovascular risk prediction. Conversely, when invasive bSBP is optimal or within normal range, oscillometric-based measurements may overestimate the BP, leading to an inappropriate diagnosis, potential unnecessary treatments, adverse effects of medications and higher costs.

### 4.3. Mean Blood Pressure: Agreement between Invasive and Non-Invasive Data (Aim 3) and Approach to Be Used with Applanation Tonometry (Aim 4)

Third and fourth, our analysis showed that (i) the best approach to estimate real (invasive) bMBP through oscillometric measurements (resulting in lower mean error) would be the equation that considers the form factor 33% (except for invasive bMBP values in the upper limit; 120 mmHg); (ii) the calibration of applanation tonometry-derived bBP waves with oscillometricbMBP (bMBP_osc_) provides the best approach to estimate the real (invasive) bSBP. Conversely, the worst association was observed when applanation tonometry-derived waves were calibrated using bMBP_033_ (bMBP = DBP + PP/3; form factor of 33%; Table 4 and Table 5, Figure 2 and Figure 3). Disregarding bMBP level, the values measured by the oscillometric device showed the greatest differences with respect to invasive bMBP. Fifth, in general terms, the differences between oscillometry-derived bMBP (regardless of the equation used) and the invasive bMBP measurements were always higher at lower bMBP levels (Figure 2B). This underlies the need for more accurate devices to measure BP, in order to minimize proportional error.

Looking at our findings, it could be said that if bMBP values are calculated in order to minimize differences with respect to invasive bMBP, the best method to use would be MBP_033_; however, when calculating BP derived from applanation tonometry, the best option would be to calibrate it with MBPosc. Our findings support the idea that “the best strategy” to calculate (quantify) bMBP depends on the aim that drives its estimation and/or on the approach and devices used. Taking into account the above, perhaps we should abandon the idea of a “false dichotomy” as to whether a given “bMBP formula” is better or worse than another, as this could vary depending on the objective pursued (and the approach considered). Our findings stress the need for methodological transparency and consensus for the non-invasive assessment of peripheral and central hemodynamic parameters. In this regard, it should be noted that, unfortunately, many studies do not describe the way in which bMBP and/or aoBP is quantified.

### 4.4. Strengths and Limitations

Our results should be analyzed in the context of the strengths and limitations of the present work. First, like most studies of this type, the work was not carried out in healthy subjects, since an invasive study is only indicated in the context of suspected or confirmed cardiovascular disease. Nevertheless, the subjects evaluated in this study are representative of the group of subjects in which the accurate knowledge of cardiovascular data is of particular clinical importance (e.g., to define the risk; to evaluate therapy).

Second, in a sample of 34 subjects we performed both a single (n = 34) analysis and an analysis based on multiple samples (n = 193) per subject. Similar and conclusive results were obtained. Although the simple analysis (n = 34) may be considered by some investigators to be carried out in an “average size n”, it should be noted that it was sufficient to detect important statistical differences, and consequently, it reached sufficient statistical power (avoiding type 2 statistical error). In addition, the concordance (agreement) of invasive and non-invasive bBP levels was estimated with several statistical methods, which increased the reliability of the findings. The invasive recordings in the contralateral brachial artery, as well as the second invasive recording at the level of the aorta, were a part of the research protocol and not of the medical diagnostic evaluation (catheterization for diagnostic purposes). The same consideration applies to the non-invasive oscillometric and tonometric recordings. The work measurements increased the duration of the catheterization by at least 30 min, limiting the number of subjects to be included in the study. Nevertheless, having 34 subjects and over 190 comparative analyses between invasive and non-invasive recordings is an important sample size for a study intended to demonstrate the relevance of several issues, but not necessarily conclusive on this important topic which will necessarily require further study.

Third, we used “fluid column” pressure transducers instead of solid-state pressure sensors. Clearly, solid-state sensors are characterized by a higher accuracy in obtaining BP waveforms, mainly because they are able to detect high-frequency components. However, fluid column transducers are widely used in clinical practice to obtain aoSBP and bSBP levels, and they are used in our University Hospital. It should be noted that in the ARTERY Society task force consensus statement on protocol standardization (“Validation of non-invasive central blood pressure devices”), Sharman et al. state that although micromanometer-tipped catheters are the preferred instruments to use, meticulously managed fluid column catheters may also be acceptable to accurately measure intra-arterial BP [22]. On the other hand, in the systematic review and meta-analysis carried out by Papaioannou et al. [1], it was reported that mean errors in the non-invasive estimation of aoSBP were similar between studies using fluid-filled and catheter-tipped transducers. Of course, compared to the high-fidelity micro-tipped catheters, the low-cost liquid-filled catheter manometer systems require more cautious handling and operation (in terms of calibration, frequency response, positioning, zeroing, etc.). However, it should also be recognized that the use of liquid-filled manometer-catheter systems (if proven accurate), should be limited only to the assessment of the maximum and minimum values of the arterial pressure waveform (as in this work), due to the damping of the wave characteristics. Conversely, in studies intended to assess the validity of the pulse waveform-derived indexes (e.g., augmented pressure or augmentation index), a high-fidelity micromanometer-tipped probe should be used to accurately assess the first systolic inflection point. In this context, considering the levels of natural frequency and damping coefficient of our catheter-tubing-external transducer system, and despite using widely validated equipment and measurement methodologies, it is clear that the invasively obtained peak systolic and minimum diastolic pressure levels may have been slightly over- and under-estimated, respectively.

Fourth, it is worth mentioning that in our work we evaluated different approaches (e.g., equations) to quantify bMBP, selecting them among the main empirical approaches (equations) proposed to estimate bMBP in the medical literature. These approaches are based on quantifying bMBP from bSBP and bDBP values, assuming different form factors (33%, 40% or 41.2%). However, despite being widely used, these equations assume that in there is a fixed relationship in each individual between bMBP, bSBP and bDBP. Additionally, the estimation of bMBP considering a fixed ratio between bDBP and bSBP may lead to a correct estimate at the population level but, unfortunately, this method may not be appropriate for estimating bMBP in the individual patient, as there are significant inter-individual variations. In this respect, recently, Grillo et al. [23] demonstrated that due to the high inter- and intra-individual variability of the pulse waveform, the estimation of bMBP based on fixed equations derived from bDBP and bSBP is not reliable. Basically, the authors demonstrated in both normotensive and hypertensive patients that in different individuals the bMBP value fluctuates widely between the bDBP and bSBP values; therefore, the relationship between bMBP and bDBP and bSBP is not fixed, predetermined or easily predictable. Secondly, they reported that there are short-term intra-individual variations in the ratio of bMBP to bSBP and bDBP. That is, in each individual, this relationship may change in relation to functional elements, such as changes in bBP or HR (e.g., stress-induced), as our own results suggested (Figure 3). Furthermore, we found that the agreement between invasive bBP and their respective non-invasive measurements (oscillometry) showed significant dependence on the bBP level. Based on this, Grillo et al. proposed that a more accurate estimation of bMBP should be ‘ideally’ based ona pulse waveform analysis, rather than on knowledge of the bSBP and bDBP values (although as they themselves mention, thisis currently difficult in daily clinical practice). In this context, future work should evaluate whether other methods of bMBP determination would allow obtaining bSBP levels closer to those recorded invasively.

Finally, as mentioned, the pattern of differences between invasive and non-invasive bBP levels could vary with age, indicating that (at least in theory) age, as well as other variables (e.g., anthropometric characteristics, sex), could be a confounding factor. Consequently, it would be important to conduct future multicenter studies with a larger number of subjects to assess the impact of age and other potentially confounding variables on the results (e.g., by performing stratification analyses by sex and/or age).

## 5. Conclusions

First, in the supine position, invasive bMBP and bDBP levels were higher in the brachial artery than in the ascending aorta.

Second, the agreement between invasive bSBP, bPP and bDBP and their respective non-invasive measurements obtained with oscillometry showed dependence on the bBP level. In general terms, the oscillometry-derived bSBP and bPP values were lower than those obtained invasively. However, invasive bSBP levels within clinically important ranges—such as “low range BP” and “high range BP values”—were overestimated and underestimated, respectively, by the non-invasive oscillometric measurements. Undoubtedly, this misestimation of BP can impair the clinical decision-making process. Only in volunteers in which bSBP was in the “normal range” was the calculated error negligible. Non-invasive oscillometric bDBP was higher than that obtained invasively without proportional error.

Third, among the different oscillometry-based approaches used to calculate bMBP, the equation that included a form factor equal to 33% was the most accurate method to estimate the real bMBP.

Fourth, the best approach to estimate invasive bSBP and bPP from brachial tonometry data is based on the calibration scheme that employs oscillometric bMBP. On the contrary, the worst association was observed when the tonometry-derived pressure wavewas calibrated with bMBP derived by bMBP = DBP + PP/3 (form factor equal to 33%).

Finally, our findings support the need for further research in the field to improve both the accuracy of the approaches used to measure BP non-invasively and the mathematical analysis to be applied to finally obtain a real central aoBP. Improved non-invasive BP assessment will, hopefully, shed light on the role of central and peripheral BP in cardiovascular health and disease.

## Figures and Tables

**Table 1:** Demographic, anthropometric and clinical characteristics (n = 34, 41% females).

Variable	MV	SE	SD	Min.	p25th	p50th	p75th	Max.	Range
Age (years)	61	3	19	14	52	68	72	89	75
Body weight (kg)	75.5	2.6	15.3	46	65	73	88	103	57
Body height (m)	166	2	9	147	162	165	174	182	35
BMI (kg/m^2^)	27.2	0.8	4.4	17.5	24.2	27.0	29.9	38.9	21.3
Hemoglobin (g/L)	12.5	1.2	2.6	8.4	12.3	12.8	13.9	15.3	6.9
Hematocrit (%)	38.1	1.8	6.6	26.0	36.0	38.3	43.0	46.0	20.0
Total cholesterol (mg/dL)	184.0	22.5	59.5	122.0	131.0	170.0	239.0	287.0	165.0
HDL cholesterol (mg/dL)	45.7	5.1	13.5	29.0	39.0	41.0	54.0	71.0	42.0
LDL cholesterol (mg/dL)	114.7	23.7	62.6	64.0	66.0	78.0	186.0	218.0	154.0
Triglycerides (mg/dL)	119.1	22.8	60.2	69.0	71.0	103.0	151.0	238.0	169.0
Atherogenic index	4.45	0.89	2.36	2.56	2.85	3.05	7.36	8.24	5.68
Creatinine (mg/dL)	1.15	0.10	0.36	0.79	0.80	1.07	1.46	1.90	1.11
Urea (mg/dL)	50.0	6.6	22.9	28.0	33.0	38.5	66.0	99.0	71.0
Glycaemia (mg/dL)	109.4	14.0	41.9	74.0	89.0	90.0	106.0	197.0	123.0
Sodium (mEq/L)	132.7	1.5	4.6	122.0	132.0	133.0	136.0	137.0	15.0
Potassium (mEq/L)	4.1	0.2	0.5	3.4	3.7	4.3	4.6	4.7	1.3
LVEDD (mm)	52.3	2.9	10.4	38.0	42.0	53.0	59.0	71.0	33.0
LVESD (mm)	31.2	2.5	8.8	18.0	25.0	30.5	37.5	45.0	27.0
LV septum thickness (mm)	10.5	0.7	2.4	6.8	8.0	11.6	12.0	14.0	7.2
LV posterior wall thickness (mm)	9.2	0.6	2.0	6.5	7.0	9.0	11.0	12.0	5.5
Left atrium area (cm^2^)	26.5	2.1	5.1	18.0	25.0	26.5	30.0	33.0	15.0
LV ejection fraction (%)	59	2	8	38	55	60	65	70	32
Active smokers (%)	5.9								
Ex-smokers (%)	48.3								
Arterial hypertension (%)	69.7								
Diabetes (%)	30.3								
Diabetics requiring insulin (%)	25.0								
Dyslipidemia (%)	60.6								
Renal insufficiency (%)	18.2								
Myocardial infarction (%)	18.2								
Acute coronary syndrome (%)	7.4								
CABG (%)	12.1								
Coronary angioplasty (%)	15.2								
ACEI (%)	37.5								
ARBs (%)	29.2								
MRAs (%)	12.5								
Beta blockers (%)	50.0								
Diuretics (%)	20.8								
Calcium channel blockers (%)	29.2								
Antiplatelet therapy (%)	31.3								
Statins (%)	66.7								
T4 (%)	8.3								

ACEI: angiotensin-converting enzyme inhibitors. ARBs: angiotensin II receptor blockers. BMI: body mass index. CABG: coronary artery bypass graft surgery. LV: leftventricle. EDD and ESD: end-diastolic and end-systolic diameter. Min. and Max.: minimum and maximal value. MRAs: Mineralocorticoid receptor antagonists. MV: mean value. SE: standard error of the mean. SD: standard deviation. p25th, p50th and p75th: 25th (first quartile), 50th (median) and 75th percentile (third quartile).

**Table 2:** Invasive and non-invasive aortic and brachial blood pressure levels.

Variable	MV	SE	SD	Min.	p25th	p50th	p75th	Max.	Range
**Invasive and non-invasive (oscillometry/plethysmography) BP recordings**									
Invasive bSBP (mmHg)	146	5	28	77	133	144	168	189	112
Invasive bMBP (mmHg)	98	3	15	66	89	98	111	122	57
Invasive bDBP (mmHg)	71	2	10	54	63	70	80	92	38
Invasive bPP (mmHg)	75	5	25	21	57	75	92	135	114
Invasive aoSBP (mmHg)	135	4	23	77	122	134	154	179	102
Invasive aoMBP (mmHg)	94	3	14	65	85	91	104	121	56
Invasive aoDBP(mmHg)	68	2	10	52	62	65	75	92	40
Invasive aoPP(mmHg)	67	4	20	22	55	64	82	103	81
Invasive HR (beat/minute)	70	3	14	49	56	68	78	104	55
bSBP (Osc) (mmHg)	137	3	19	85	127	135	156	167	82
bDBP (Osc) (mmHg)	81	2	13	55	73	79	90	108	53
bPP (Osc) (mmHg)	56	3	14	29	45	55	67	91	62
HR (Osc) (mmHg)	71	3	14	44	59	72	81	105	61
bMBP_0.42_(mmHg)	105	3	14	68	96	106	112	130	62
bMBP_0.412_(mmHg)	104	2	14	68	96	106	111	130	62
bMBP_0.33_(mmHg)	100	2	13	65	92	100	106	125	60
bMBP_+5_(mmHg)	105	2	13	70	97	105	111	130	59
bMBP_0.33HR_(mmHg)	104	3	14	68	96	104	110	131	63
bMBP_SBP×DBP_^0.5^ (mmHg)	105	3	14	69	97	106	112	132	63
bMBP_osc_(mmHg)	107	3	14	69	98	108	115	133	64
**Invasive and non-invasive (applanation tonometry) BP recordings**									
Invasive bSBP (mmHg)	145	5	27	85	133	144	166	189	104
Invasive bMBP (mmHg)	98	3	15	68	88	98	110	120	52
Invasive bDBP (mmHg)	70	2	10	53	63	69	77	91	38
Invasive bPP (mmHg)	75	4	24	25	61	75	89	136	111
Invasive aoSBP (mmHg)	134	4	25	77	121	130	154	179	102
Invasive aoMBP (mmHg)	94	3	15	65	85	91	106	121	56
Invasive aoDBP(mmHg)	68	2	10	52	62	65	75	92	40
Invasive aoPP(mmHg)	66	4	21	22	54	64	82	103	81
Invasive HR (beat/minute)	70	3	15	50	58	68	78	104	54
bSBP (Osc) (mmHg)	136	4	21	78	126	136	150	167	89
bDBP (Osc) (mmHg)	81	3	15	43	74	79	92	105	62
bPP (Osc) (mmHg)	55	2	14	28	45	53	66	83	55
HR (Osc) (mmHg)	71	2	14	44	61	71	81	104	60
bMBP_0.42_(mmHg)	104	3	16	58	97	105	111	130	72
bMBP_0.412_(mmHg)	103	3	16	57	96	105	110	129	72
bMBP_0.33_(mmHg)	99	3	16	55	92	100	106	124	70
bMBP_+5_(mmHg)	104	3	16	60	97	105	111	129	70
bMBP_0.33HR_(mmHg)	104	3	16	57	96	105	111	131	74
bMBP_SBP×DBP_^0.5^ (mmHg)	104	3	16	58	97	106	111	131	73
bMBP_osc_(mmHg)	106	3	16	59	99	108	114	133	74
bSBP (AT_SD) (mmHg)	136	4	21	78	126	136	150	167	89
bDBP (AT_SD) (mmHg)	81	3	15	43	74	79	92	105	62
bMBP (AT_SD) (mmHg)	104	3	18	52	96	104	116	133	81
bSBP (AT_033) (mmHg)	124	3	18	73	116	125	135	158	85
bDBP (AT_033) (mmHg)	81	3	15	43	74	79	92	105	62
bMBP (AT_033) (mmHg)	99	3	16	54	92	100	106	124	70
bSBP (AT_033HR) (mmHg)	136	3	20	79	125	136	150	175	96
bDBP (AT_033HR) (mmHg)	81	3	15	43	74	79	92	105	62
bMBP (AT_033HR) (mmHg)	103	3	16	57	96	105	111	131	74
bSBP (AT_0412) (mmHg)	136	3	19	79	126	136	150	172	93
bDBP (AT_0412) (mmHg)	81	3	15	43	74	79	92	105	62
bMBP (AT_0412) (mmHg)	103	3	16	57	96	105	110	129	72
bSBP (AT_Osc) (mmHg)	142	4	21	81	131	140	157	180	99
bDBP (AT_Osc) (mmHg)	81	3	15	43	74	79	92	105	62
bMBP (AT_Osc) (mmHg)	106	3	16	59	99	107	114	133	74
bSBP (AT_inv) (mmHg)	136	4	23	80	123	143	151	181	101
bDBP (AT_Inv) (mmHg)	70	2	10	53	63	69	77	91	38
bMBP (AT_Inv) (mmHg)	98	3	15	68	88	98	110	120	52

Prefixes ‘b’ and ‘ao’ indicate brachial artery and aorta, respectively. HR: heart rate; MV: mean value; SBP, MBP, DBP and PP: systolic, mean, diastolic and pulse blood pressure, respectively; SD: standard deviation; SE: standard error of the mean; p25th, p50th and p75th: 25th percentile (first quartile), 50th percentile (median) and 75th percentile (third quartile).

**Table 3:** Comparison between invasive brachial artery and aortic blood pressure.

	SBP	MBP	DBP	PP
**Lin’s Concordance Correlation Coefficient (CCC) (Aortic: x, Brachial: y) (n = 34)**				
CCC	0.79	0.87	0.85	0.78
CCC, 95% CI	0.63 to 0.88	0.76 to 0.94	0.72 to 0.92	0.63 to 0.88
R	0.87	0.90	0.87	0.86
r, 95% CI	0.74 to 0.94	0.81 to 0.95	0.75 to 0.94	0.73 to 0.93
*p* value	<0.0001	<0.0001	<0.0001	<0.0001
**Intraclass Correlation Coefficient (ICC) (n = 34)**				
ICC: Single measures	0.79	0.88	0.86	0.79
ICC, 95% CI	0.40 to 0.92	0.68 to 0.98	0.70 to 0.93	0.49 to 0.91
ICC: Average measures	0.88	0.93	0.92	0.88
ICC, 95% CI	0.57 to 0.96	0.81 to 0.97	0.82 to 0.96	0.66 to 0.95
**Bland–Altman Test (Reference: brachial artery record) (n = 34)**				
Mean error (mmHg)	10.46	3.79	2.31	8.17
95% CI (mean error) (mmHg)	5.45 to 15.48	1.47 to 6.12	0.39 to 4.22	3.42 to 12.93
*p* (H_0_: Mean = 0)	<0.0001	0.002	0.020	0.001
Lower limit (mmHg)	−16.36	−8.63	−7.93	−17.25
Upper limit (mmHg)	37.29	16.22	12.54	33.60
Regression Equation	y = 5.965 + 0.0333x	y = 9.943 − 0.0651x	y = 12.027 − 0.143x	y = 0.867 + 0.109x
Intercept, *p* value	0.694	0.213	0.060	0.919
Slope, *p* value	0.764	0.432	0.120	0.375
**Bland–Altman Test: multiple measurements per subject (reference: brachial artery record) (n = 193)**				
Mean error (mmHg)	10.66	3.90	2.33	8.33
Lower limit (mmHg)	−16.61	−9.33	−9.17	−18.21
Upper limit (mmHg)	37.93	17.13	13.82	34.87

CCC: Concordance Correlation Coefficient. ICC: Intraclass Correlation Coefficient. r: Pearson coefficient. CI: Confidence Interval.

**Table 4:** Comparison of invasive and non-invasive (oscillometry) brachial blood pressure levels.

	bSBP	bDBP	bPP	bMBP_042_	bMBP_0.412_	bMBP_033_	bMBP_+5_	bMBP_0.33HR_	bMBP_SBP*DBP_^0.5^	bMBP_Osc_
**Lin’s Concordance Correlation Coefficient (CCC) (Non-invasive: x, Invasive: y) (n = 34)**										
CCC	0.77	0.39	0.42	0.74	0.75	0.80	0.73	0.76	0.73	0.70
95% CI (LL/UL)	0.63/0.86	0.16/0.56	0.23/0.59	0.56/0.86	0.57/0.87	0.63/0.90	0.53/0.85	0.57/0.87	0.53/0.85	0.51/0.83
R	0.88	0.58	0.70	0.82	0.82	0.81	0.80	0.82	0.81	0.83
95% CI (LL/UL)	0.76/0.94	0.28/0.77	0.46/0.84	0.66/0.91	0.66/0.91	0.63/0.90	0.63/0.90	0.66/0.91	0.64/0.91	0.67/0.91
*p*	<0.0001	<0.0001	<0.0001	<0.0001	<0.0001	<0.0001	<0.0001	<0.0001	<0.0001	<0.0001
**Intraclass Correlation Coefficient (ICC) (n = 34)**										
Single Meas	0.77	0.4	0.43	0.75	0.76	0.80	0.73	0.76	0.73	0.71
95% CI	0.50/0.90	−0.05/0.70	−0.05/0.72	0.36/0.89	0.41/0.89	0.63/0.90	0.36/0.88	0.40/0.90	0.30/0.89	0.11/0.89
Average Meas	0.87	0.57	0.6	0.86	0.86	0.89	0.85	0.86	0.85	0.83
ICC, 95% CI	0.67/0.94	−0.10/0.82	−0.11/0.84	0.53/0.94	0.58/0.94	0.78/0.95	0.52/0.94	0.57/0.95	0.46/0.94	0.20/0.94
**Bland–Altman Test (reference: non-invasive recording) (n = 34)**										
ME (mmHg)	−8.2	10.6	−18.8	6.3	5.9	1.4	6.3	6.0	6.9	8.5
95% CI (mmHg)	−13.4/−3.0	6.6/14.5	−25.5/−12.1	3.2/9.5	2.7/9.0	−1.8/4.7	3.0/9.5	2.9/9.2	3.6/10.1	5.4/11.6
*p* (H_0_: Mean = 0)	<0.0001	<0.0001	<0.0001	<0.0001	<0.0001	0.374	<0.0001	<0.0001	<0.0001	<0.0001
LL (mmHg)	−36.2	−10.8	−54.6	−10.5	−11	−16	−11.2	−10.8	−10.5	−8.1
UL (mmHg)	19.8	31.9	17.1	23.2	22.8	18.8	23.7	22.9	24.2	25.1
Eq. (y=)	50.01 − 0.40x	29.98 − 0.27x	26.06 − 0.59x	28.26 − 0.22x	28.08 − 0.22x	27.17 − 0.26x	32.23 − 0.26x	26.33 − 0.20x	28.94 − 0.22x	28.70 − 0.20x
Intercept, *p*	<0.0001	0.0348	0.0002	0.0082	0.0086	0.0111	0.0032	0.0141	0.0088	0.0073
Slope, *p*	<0.0001	0.158	<0.0001	0.0339	0.0318	0.0146	0.0141	0.0508	0.0387	0.049
**Bland–Altman Test: multiple measurements per subject (reference: non-invasive recording) (n = 193)**										
ME (mmHg)	−8.4	10.5	−18.8	6.3	5.9	1.5	6.3	6.1	6.9	8.5
LL (mmHg)	−39	−13.2	−57.2	−12.8	−13.2	−18.3	−13.5	−13	−12.9	−10.4
UL (mmHg)	22.3	34.2	19.5	25.4	25	21.2	26.1	25.2	26.7	27.4

CCC: ConcordanceCorrelation Coefficient. ICC: Intraclass Correlation Coefficient. r: Pearson coefficient. SD and SE: Standard deviation and error, respectively. 95% CI: 95% Confidence Interval. ME: mean error. LL: lower limit. UL: upper limit. *p*: *p* value. Meas: measurement. Eq.: Equation. Prefix “b” indicate brachial artery. Brachial MBP (bBP) was obtained using oscillometry (osc) and six different equations (see Figure 1 and text).

**Table 5:** Comparison between invasive and non-invasive (applanation tonometry) brachial blood pressure levels.

	Calibration Scheme:											
	bSBP/bDBP		bMBP_0.33_/bDBP		bMBP_0.33HR_/bDBP		bMBP_0.412_/bDBP		bMBP_osc_/bDBP		bMBPinv/bDBPinv	
	bSBP	bPP	bSBP	bPP	bSBP	bPP	bSBP	bPP	bSBP	bPP	bSBP	bPP
**Lin’s Concordance Correlation Coefficient (CCC) (Non-invasive: x, Invasive: y) (n = 34)**												
CCC	0.77	0.38	0.43	0.13	0.62	0.26	0.60	0.24	0.64	0.30	0.81	0.75
95% CI (LL/UP)	0.62/0.86	0.19/0.54	0.23/0.59	0.01/0.25	0.38/0.77	0.03/0.45	0.36/0.76	0.02/0.43	0.39/0.80	0.02/0.53	0.66/0.89	0.58/0.85
R	0.89	0.70	0.71	0.40	0.71	0.43	0.70	0.40	0.67	0.39	0.88	0.86
r, 95% CI	0.77/0.94	0.45/0.84	0.47/0.85	0.04/0.66	0.47/0.85	0.08/0.68	0.44/0.8	0.04/0.66	0.41/0.83	0.03/0.65	0.76/0.94	0.72/0.93
*p*	<0.0001	<0.0001	<0.0001	0.0307	<0.0001	0.0172	<0.0001	0.0294	<0.0001	0.0335	<0.0001	<0.0001
**Intraclass Correlation Coefficient (ICC) (n = 34)**												
Single Meas	0.78	0.39	0.44	0.14	0.63	0.26	0.61	0.24	0.65	0.31	0.82	0.76
ICC, 95% CI	0.38/0.90	−0.09/0.71	−0.08/0.74	−0.09/0.41	0.30/0.81	−0.08/0.56	0.28/0.80	−0.08/0.54	0.38/0.81	−0.03/0.58	0.42/0.93	0.32/0.90
Average Meas	0.87	0.56	0.61	0.24	0.77	0.42	0.76	0.39	0.79	0.47	0.90	0.86
ICC, 95% CI	0.56/0.95	−0.21/0.83	−0.18/0.85	−0.20/0.59	0.46/0.90	−0.17/0.72	0.44/0.89	−0.18/0.70	0.56/0.90	−0.05/0.74	0.60/0.96	0.49/0.95
**Bland–Altman Test (reference: non-invasive recording) (n = 34)**												
ME [mmHg]	−9.5	−20.9	−21.6	−33.0	−10.0	−21.6	−9.9	−21.4	−4.0	−15.4	−9.6	−9.6
95% CI [mmHg]	−14.3/−4.7	−27.2/−14.6	−28.4/−14.7	−41.1/−24.7	−16.8/−3.2	−29.7/−13.3	−16.9/−2.9	−29.9/−12.9	−11.2/3.3	−24.2/−6.5	−14.1/−5.0	−14.1/−5.0
*p* (H_0_: Mean = 0)	<0.0001	<0.0001	<0.0001	<0.0001	0.0053	<0.0001	0.007	<0.0001	0.274	0.0013	0.0002	0.0002
LL [mmHg]	−34.5	−54.2	−57.5	−76.0	−45.8	−64.8	−46.6	−65.9	−42.2	−61.7	−33.4	−33.5
UL [mmHg]	15.5	12.3	14.4	10.1	25.7	21.7	26.7	23.0	34.3	30.9	14.3	14.2
Eq. (y =)	43.40 − 0.36x	23.95 − 0.58x	57.99 − 0.54x	26.55 − 0.77x	59.74 − 0.47x	32.13 − 0.69x	63.07 − 0.49x	33.97 − 0.72x	65.67 − 0.47x	37.67 − 0.69x	25.07 − 0.23x	15.56 − 0.32x
Intercept, *p*	<0.0001	<0.0001	<0.0001	0.0030	<0.0001	0.0022	<0.0001	0.0016	<0.0001	0.0002	0.0353	0.0161
Slope, *p*	<0.0001	<0.0001	<0.0001	<0.0001	<0.0001	<0.0001	<0.0001	<0.0001	<0.0001	<0.0001	0.0043	<0.0001
**Bland–Altman Test: multiple measurements per subject (reference: non-invasive recording) (n= 128)**												
ME [mmHg]	−9.4	−20.8	−21.4	−32.8	−9.9	−21.4	−9.8	−21.3	−3.8	−15.2	−9.5	−9.5
LL [mmHg]	−36.3	−55.6	−59.5	−77.4	−48.1	−66.5	−48.8	−67.5	−44.6	−63.4	−35.6	−35.4
UL [mmHg]	17.4	14.1	16.6	11.7	28.3	23.7	29.2	25.0	36.9	33.0	16.7	16.5

Prefix “b” indicates brachial artery. Brachial MBP was obtained using oscillometry (osc) and six different equations (see text). 95% CI: 95% Confidence Interval. CCC: Concordance Correlation Coefficient. ICC: Intraclass Correlation Coefficient. inv: invasive.LL: lower limit.osc: oscillometry.ME: mean error.r: Pearson coefficient. SBP, MBP, DBP and PP: systolic, mean, diastolic and pulse blood pressure, respectively. Meas: measurement. Eq.: Equation. UL: upper limit.

## Data Availability

Not applicable.

## Abbreviations

aoBP	Central aortic blood pressure
aoMBP	Central aortic mean blood pressure
aoSBP	Central aortic systolic blood pressure
bBP	Brachial artery blood pressure
bDBP	Brachial artery diastolic blood pressure
bDBP(AT_033)	Brachial artery diastolic blood pressure (‘bDBP’) obtained with applanation tonometry (‘AT’; SphygmoCor device), calibrating with bMBP_0.33_ and bDBP (Oscillometry)
bDBP(AT_033HR)	Brachial artery diastolic blood pressure (‘bDBP’) obtained with applanation tonometry (‘AT’; SphygmoCor device), calibrating with bMBP_0.33HR_ and bDBP (Oscillometry).
bDBP(AT_0412)	Brachial artery diastolic blood pressure (‘bDBP’) obtained with applanation tonometry (‘AT’; SphygmoCor device), calibrating with bMBP_0.412_ and bDBP (Oscillometry).
bDBP(AT_Inv)	Brachial artery diastolic blood pressure (‘bDBP’) obtained with applanation tonometry (‘AT’; SphygmoCor device), calibrating with invasive (‘Inv’) bMBP and bDBP
bDBP(AT_Osc)	Brachial artery diastolic blood pressure (‘bDBP’) obtained with applanation tonometry (‘AT’; SphygmoCor device), calibrating with bMBP (Oscillometry) and bDBP (Oscillometry).
bDBP(AT_SD)	Brachial artery diastolic blood pressure (‘bDBP’) obtained with applanation tonometry (‘AT’; SphygmoCor device), calibrating with bSBP (Oscillometry) and bDBP (Oscillometry).
bDBP(Osc)	Brachial artery diastolic blood pressure obtained with the oscillometric system (Mobil-O-Graph device).
bMBP	Brachial artery mean blood pressure
bMBP(AT_033)	Brachial artery mean blood pressure (‘bMBP’) obtained with applanation tonometry (‘AT’; SphygmoCor device), calibrating with bMBP_0.33_ and bDBP (Oscillometry).
bMBP(AT_033HR)	Brachial artery mean blood pressure (‘bMBP’) obtained with applanation tonometry (‘AT’; SphygmoCor device), calibrating with bMBP_0.33HR_ and bDBP (Oscillometry).
bMBP(AT_0412)	Brachial artery mean blood pressure (‘bMBP’) obtained with applanation tonometry (‘AT’; SphygmoCor device), calibrating with bMBP_0.412_ and bDBP (Oscillometry).
bMBP(AT_Inv)	Brachial artery mean blood pressure (‘bMBP’) obtained with applanation tonometry (‘AT’; SphygmoCor device), calibrating with invasive (‘Inv’) bMBP and bDBP
bMBP(AT_Osc)	Brachial artery mean blood pressure (‘bMBP’) obtained with applanation tonometry (‘AT’; SphygmoCor device), calibrating with bMBP (Oscillometry) and bDBP (Oscillometry).
bMBP(AT_SD)	Brachial artery mean blood pressure (‘bMBP’) obtained with applanation tonometry (‘AT’; SphygmoCor device), calibrating with bSBP (Oscillometry) and bDBP (Oscillometry).
bMBPosc	Brachial artery mean blood pressure obtained with the oscillometric system (Mobil-O-Graph device).
bPP	Brachial artery pulse pressure
bPP(Osc)	Brachial artery pulse pressure obtained with the oscillometric system (Mobil-O-Graph device).
bSBP	Brachial artery systolic blood pressure
bSBP(AT_033)	Brachial artery systolic blood pressure (‘bSBP’) obtained with applanation tonometry (‘AT’; SphygmoCor device), calibrating with bMBP_0.33_ and bDBP (Oscillometry).
bSBP(AT_033HR)	Brachial artery systolic blood pressure (‘bSBP’) obtained with applanation tonometry (‘AT’; SphygmoCor device), calibrating with bMBP_0.33HR_ and bDBP (Oscillometry).
bSBP(AT_0412)	Brachial artery systolic blood pressure (‘bSBP’) obtained with applanation tonometry (‘AT’; SphygmoCor device), calibrating with bMBP_0.412_ and bDBP (Oscillometry).
bSBP(AT_Inv)	Brachial artery systolic blood pressure (‘bSBP’) obtained with applanation tonometry (‘AT’; SphygmoCor device), calibrating with invasive (‘Inv’) bMBP and bDBP.
bSBP(AT_Osc)	Brachial artery systolic blood pressure (‘bSBP’) obtained with applanation tonometry (‘AT’; SphygmoCor device), calibrating with bMBP (Oscillometry) and bDBP (Oscillometry).
bSBP(AT_SD)	Brachial artery systolic blood pressure (‘bSBP’) obtained with applanation tonometry (‘AT’; SphygmoCor device), calibrating with bSBP (Oscillometry) and bDBP (Oscillometry).
bSBP(Osc)	Brachial artery systolic blood pressure obtained with the oscillometric system (Mobil-O-Graph device).
CCC	Lin’s Concordance Correlation Coefficient
CRFs	Cardiovascular risk factors
HR	Heart rate
HR(Osc)	Heart rate obtained with the oscillometric system (Mobil-O-Graph device).
95% CI	95% Confidence Interval
ICC	Intraclass Correlation Coefficient
MBP	Mean blood pressure
